# Identification of *SEC62* as a potential marker for 3q amplification and cellular migration in dysplastic cervical lesions

**DOI:** 10.1186/s12885-016-2739-6

**Published:** 2016-08-23

**Authors:** Maximilian Linxweiler, Florian Bochen, Bernhard Schick, Silke Wemmert, Basel Al Kadah, Markus Greiner, Andrea Hasenfus, Rainer-Maria Bohle, Ingolf Juhasz-Böss, Erich-Franz Solomayer, Zoltan Ferenc Takacs

**Affiliations:** 1 Department of Otorhinolaryngology, Saarland University Medical Center, Kirrberger Street 100, Building 6, 66421 Homburg/Saar, Germany; 2 Department of Medical Biochemistry and Molecular Biology, Saarland University Medical Center, Kirrberger Street 100, Building 44, Homburg/Saar, Germany; 3 Department of General and Surgical Pathology, Saarland University Medical Center, Kirrberger Street 100, Building 26, Homburg/Saar, Germany; 4 Department of Gynecology, Obstetrics and Reproductive Medicine, Saarland University Medical Center, Kirrberger Street 100, Building 9, Homburg/Saar, Germany

**Keywords:** *SEC62*, 3q amplification, Cervical dysplasia, Cell migration, Epithelial-mesenchymal transition

## Abstract

**Background:**

Chromosome 3 amplification affecting the 3q26 region is a common genomic alteration in cervical cancer, typically marking the transition of precancerous intraepithelial lesions to an invasive phenotype. Though potential 3q encoded target genes of this amplification have been identified, a functional correlation of potential oncogenic function is still missing. In this study, we investigated copy number changes and the expression level of *SEC62* encoded at 3q26.2 as a new potential 3q oncogene in dysplastic cervical lesions and analyzed its role in cervical cancer cell biology.

**Methods:**

Expression levels of Sec62 and vimentin were analyzed in liquid based cytology specimens from 107 women with varying grades of cervical dysplasia ranging from normal cases to cancer by immunofluorescence cytology. Additionally, a subset of 20 representative cases was used for FISH analyses targeting *SEC62*. To further explore the functional role of Sec62 in cervical cancer, HeLa cells were transfected with a *SEC62* plasmid or *SEC62* siRNA and analyzed for their proliferation and migration potential using real-time monitoring and trans-well systems as well as changes in the expression of EMT markers.

**Results:**

FISH analyses of the swabbed cells showed a rising number of *SEC62* gains and amplifications correlating to the grade of dysplasia with the highest incidence in high grade squamous intraepithelial lesions and squamous cell carcinomas. When analyzing the expression level of Sec62 and vimentin, we found a gradually increasing expression level of both proteins according to the severity of the dysplasia. In functional analyses, *SEC62* silencing inhibited and *SEC62* overexpression stimulated the migration of HeLa cells with only marginal effects on cell proliferation, the expression level of EMT markers and the cytoskeleton structure.

**Conclusions:**

Our study suggests *SEC62* as a target gene of 3q26 amplification and a stimulator of cellular migration in dysplastic cervical lesions. Hence, *SEC62* could serve as a potential marker for 3q amplification, providing useful information about the dignity and biology of dysplastic cervical lesions.

**Electronic supplementary material:**

The online version of this article (doi:10.1186/s12885-016-2739-6) contains supplementary material, which is available to authorized users.

## Background

Cervical cancer represents the third most common cancer in women worldwide and accounts for approximately 8 % of all female cancer deaths [1]. Over the past decades, the molecular carcinogenesis of this cancer entity has been intensively studied. This has not only led to a better understanding of cancer cell biology, but also resulted in new therapeutic approaches, e.g., the clinical use of Bevacizumab in advanced and recurrent cases of cervical cancer [2]. An amplification of the long arm of chromosome 3 (3q) has been identified as a characteristic genomic alteration in more than 75 % of cervical cancer cases [3, 4] and the smallest amplified region was mapped down to 3q26-27 [5, 6]. When screening dysplastic cells of precancerous cervical lesions for this genomic alteration, the frequency of 3q amplification increased with the severity of the dysplasia with an incidence of 8–35 % in severe dysplasia [7] and 32–90 % in invasive squamous cell carcinomas [3, 4, 8, 9]. In normal cervical epithelium as well as mild and moderate dysplasia, 3q amplification was only sporadically found [7]. Thus, 3q amplification designates the transition from intraepithelial cervical neoplasia to invasive cancer [3].

Apart from cervical cancer, 3q amplification was identified as a common genomic alteration in other cancers as well including non-small-cell lung cancer (NSCLC) [10], esophageal cancer [11], ovarian cancer [12] and head and neck squamous cell carcinomas (HNSCC) [13, 14]. Consequently, much effort has been spent identifying potential oncogenes encoded in this region. This has led to the identification of *SEC62* [15], *PIK3CA* [16], *SOX2* [17], *TP63* [18], *EIF4G*, *CLAPM1* and *FXR1* [19] as candidate oncogenes, but no functional correlation of potential oncogenic function has been reported for the majority of these genes. However, for *SEC62* encoding for an endoplasmic reticulum transmembrane protein involved in intracellular protein transport [20–22], we previously reported that overexpression of *SEC62* increases the migration ability of different human cancer cells as a basic mechanism of metastasis [15, 23]. These data suggest *SEC62* as a migration-stimulating oncogene [24]. Nevertheless, the molecular mechanism of migration stimulation by the *SEC62 gene* remains unknown. In this context, a recent proteomic study demonstrated that stable overexpression of *SEC62* in HEK293 cells induced a rise in vimentin expression [25] and a morphological change of the actin cytoskeleton. Consequently, it was proposed that the *SEC62*-induced stimulation of cell migration could be mediated by the induction of epithelial-mesenchymal transition (EMT).

EMT, a highly conserved biological process leading to the induction of invasive growth and metastasis formation, has intensively been studied and is described for multiple cancers, including cervical cancer [26–28]. On the molecular level, EMT is marked by an increased expression of vimentin, a reorganization of the actin cytoskeleton and downregulation of E-cadherin with a switch to higher levels of N-cadherin [29, 30]. In cervical cancer, epidermal growth factor (EGF) has been shown to be a potent inducer of EMT and to be associated with tumor invasion and lymph node metastases [31, 32].

In this study, we investigated (i) if 3q amplification in precancerous and cancerous cervical lesions targets *SEC62* as potential 3q encoded oncogene, (ii) if the dysplastic cervical cells show a corresponding overexpression of the *SEC62* gene and (iii) if *SEC62* had an oncogenic function in cultured cervical cancer cells through altering cell migration, cell proliferation and EMT induction.

## Methods

### Patient characteristics and liquid-based cytology

In total, 107 female patients were enrolled in this study who presented at the Department of Gynecology, Obstetrics and Reproductive Medicine of the Saarland University Medical Center (Homburg/Saar, Germany) between January 2012 and January 2013 in the context of the national cervical cancer prevention program. From all patients, liquid-based cytological swab material of the uterine cervix was used for further analyses. Thereby, we collected subsamples for cytological negative samples, and each of the histology groups CIN-I (cervical intraepithelial lesion grade I) through CIN-III (cervical intraepithelial lesion grade III; each of size 25) as well as a sample of 7 patients with histologic SCC (squamous cell carcinoma). For 82 patients (82/107; 76.6 %), probe excisions of the uterine cervix were also available. For patients with a normal cytological swab, we abstained from an incisional biopsy. Exclusion criteria included a history of surgical or medicinal treatment of dysplastic cervical lesions, an acute or chronic cervicitis or colpitis and non representative cytological or histological material. From each patient, a cytological smear from the uterine cervix was taken using the Cytobrush Plus (Cooper Surgical Inc.; Trumbull, CT, USA) in an ambulatory setting. After wiping off the macroscopically suspect mucosal areas, brushes were shaken out in the PreservCyt solution (Hologic Deutschland GmbH; Wiesbaden, Germany). The cellular suspensions were used for the preparation of microscope slides using the ThinPrep-system (Hologic Deutschland GmbH; Wiesbaden, Germany) according to the manufacturer’s instructions. For cytopathological staging, the microscope slides were stained according to Papanicolaou using a standard protocol. The slides were classified by two independent examiners with wide experience in valuing cytological smears of the uterine cervix. The respective cytological diagnoses according to the Bethesda classification system were NILM (negative for intraepithelial lesion/malignancy, *n* = 25), ASCUS (atypical squamous cells of undetermined significance, *n* = 9), LSIL (low-grade squamous intraepithelial lesion, *n* = 25), HSIL (high-grade squamous intraepithelial lesion, *n* = 38) and SCC (squamous cell carcinoma, *n* = 10). The Saarland Medical Association ethics review committee approved the scientific use of the patient’s tissue and clinical data (index number 207/10). Written informed consent was obtained from all patients.

### Fluorescence in situ hybridization (FISH) analysis

Prepared microscope slides were pretreated with RNase A and pepsin, then denatured with 70 % formamide/2xSSC at 72 °C, dehydrated in a series of cold ethanol washes and air-dried.

The BAC clone RP11-379 K17 encoding *SEC62* (ImaGenes, Berlin, Germany) was biotin labeled using the BioPrime DNA Labeling System (Invitrogen, Life Technologies, Darmstadt, Germany). As internal control, a centromeric probe for chromosome 10 (D10Z3) labeled with digoxigenin by standard nick-translation according to the manufacturer’s instructions (Roche Diagnostics GmbH, Mannheim, Germany) was used. After probe hybridization overnight, the slides were washed two times in 2× SSC at 42 °C and three times in 50 % formamide/2× SSC at 42 °C. Immunofluorescence detection of the biotin signals was carried out using Streptavidin-FITC and -biotinylated anti-Streptavidin antibodies (Vector Laboratories, Burlingame, CA, USA). For the detection of the digoxigenin signals, anti-Dig-Cy3 and goat-anti-mouse-Cy3 (Jackson ImmunoResearch Laboratories, West Grove, PA, USA) were used. The slides were mounted in an anti-fade solution containing DAPI (4, 6-diamidino-2-phenylindole; Vector Laboratories, Burlingame, CA, USA) and analyzed with the BX61 fluorescent microscope equipped with a charge-coupled device camera (Olympus, Hamburg, Germany). In total, 200 non-overlapping, morphologically well-preserved nuclei per slide were analyzed. Thereby, we selectively evaluated the number of FISH signals in the morphologically conspicuous nuclei in the CIN-I, CIN-II, CIN-III and SCC (histological diagnosis) cases. For the “no CIN” cases, every nucleus was considered. Gains were defined as three or four signals per probe; five or more signals were defined as amplification. The specificity of each probe was determined by hybridizing and enumerating normal human lymphocytes and metaphase spreads, prepared according to standard protocols, for cutoff ranges and an analysis of cross hybridizations by non-stringency of hybridization conditions.

FISH analyses were performed on cytological specimens in a representative subset of 20 patients with histological diagnoses of “no CIN” (*n* = 5; cytological diagnosis NILM [*n* = 5]), CIN-I (*n* = 5; cytological diagnosis ASCUS [*n* = 1], LSIL [*n* = 3] and HSIL [*n* = 1]), CIN-II (*n* = 5; cytological diagnosis ASCUS [*n* = 1] and HSIL [*n* = 4]), CIN-III (*n* = 5, cytological diagnosis HSIL [*n* = 4] and SCC [*n* = 1]) and SCC (*n* = 5; cytological diagnosis SCC [*n* = 5]).

### Immunofluorescence cytology (IFC)

To simultaneously analyze Sec62 and vimentin expression in the swabbed cells, prepared microscope slides were dried for 30 min at room temperature. The slides were washed three times in distilled water (aqua dest.) and PBS pH7.2. Epitope unmasking was performed by incubation in Target Retrieval Solution (DAKO, Glostrup, Denmark) at 95 °C for 60 min. After cooling to room temperature and three PBS pH 7.2 washes, the slides were incubated with the primary antibody solution (1:100 dilution in 0.1 % BSA/PBS) for 60 min at room temperature. After another three PBS washes, the slides were incubated with the secondary antibody solution (1:100 dilution in 0.1 % BSA/PBS) for 60 min at room temperature, again followed by three PBS washes. The slides were counterstained with Hemalaun (1:4 dilution in aqua dest.) and mounted in DAPI-Fluoroshield -mounting medium (Sigma-Aldrich, St. Louis, MO, USA).

To detect Sec62, we generated a polyclonal affinity-purified rabbit antibody directed against the COOH-terminal undecapeptide of the human Sec62 protein as previously described [15, 23–25] and detected it with a goat anti-rabbit secondary antibody conjugated with fluorescein isothiocyanate (FITC; Dianova, Hamburg, Germany). The monoclonal Clone 9 vimentin antibody was labeled with Cy3 (Sigma-Aldrich, St. Louis, MO, USA). Slides were imaged with the Nikon Eclipse TE2000-S inverted microscope, the Nikon Digital Sight DS-5Mc camera and the NIS-Elements AR software version 3.0 (Nikon; Tokyo, Japan).

The fluorescent signals for Sec62 and vimentin were quantified in morphologically dysplastic cells in relation to normal cells of the same slide by six independent examiners. The staining intensity was valued as “-1“for a weaker fluorescent signal in dysplastic cells compared with normal cells, “0” for no difference in the staining intensity between dysplastic and normal cells and “+1”, “+2” or “+3” for a little stronger, moderately stronger or markedly stronger signals in dysplastic cells compared with normal cells. If no dysplastic cells were found in the slide, the staining intensity of two normal cells was compared to each other. The overall immunoreactive score (IRS) for Sec62 and vimentin was set as a sum of the six single scorings (six separate examiners) with a minimal score of −6 and a maximal score of 18. For all IFC analyses, we referred to the histological diagnosis when grouping the patients into the CIN-I, CIN-II, CIN-III and SCC group. For the “no CIN” cases we had to refer to the cytological diagnosis as no probe excision of the uterine cervix was available for these patients.

### Cell culture and transfections

HeLa cells (DSMZ-No. ACC 57) and MCF-7 cells (DSMZ-No. ACC 115) were cultured in DMEM medium (Gibco Invitrogen, Karlsruhe, Germany) containing 10 % FBS (Biochrom, Berlin, Germany) and 1 % penicillin/streptomycin (PAA, Pasching, Austria) at 37 °C in a humidified environment with 5 % CO_2_. Both cell lines were characterized by the German Collection of Microorganisms and Cell Culture (DSMZ) using multiplex PCR of minisatellite markers, isoelectric focusing and karyotyping. The cell lines were obtained by the DSMZ in 2015.

For gene silencing, 5.2 × 10^5^ HeLa cells were seeded in 6 cm dishes and transfected with *SEC62* siRNA directed against the 3′ untranslated region (CGUAAAGUGUAUUCUGUACtt; Ambion, TX, USA) or control siRNA (AllStars Neg. control siRNA; Qiagen, Hilden, Germany) using HiPerFect Transfection Reagent (Qiagen, Hilden, Germany) according to the manufacturer’s instructions. After 24 h, the medium was changed and the cells were transfected again for additional 24 h.

For overexpression studies, 5.2 × 10^5^ HeLa cells were seeded in 6 cm dishes. After 24 h, the medium was changed and the cells were transfected with either the IRES-*GFP*-*SEC62* plasmid (*SEC62* plasmid) or the negative control IRES-*GFP*-LV plasmid (control plasmid) using X-tremeGENE HP DNA Transfection Reagent (Roche Diagnostics GmbH, Mannheim, Germany) according to the manufacturer’s instructions. For both plasmids, pcDNA3 served as parent plasmid.

### Western blot

2 × 10^5^ HeLa cells were lysed in a lysis buffer (aqua dest. + 10 mM NaCl/10 mM Tris(hydroxymethyl)-aminomethan/3 mM MgCl_2_/5 % NP-40) and proteins were resolved by SDS-PAGE and identified by immunoblotting. Antibodies used were the previously described anti-human Sec62, monoclonal anti-human β-actin (Sigma-Aldrich Co., St. Louis, MO, USA), anti-human E-cadherin Clone 24E10 (Cell signaling Technology, Cambridge, UK), anti-human vimentin Clone V9 (Dako Denmark A/S, Glostrup, Denmark) and anti-human GAPDH (sc-25778, Santa Cruz Biotechnology, Dallas, TX, USA) antibody. Secondary antibodies used were ECL Plex goat anti-rabbit Cy5 or anti-mouse Cy3 conjugates (GE Healthcare, Munich, Germany). Blots were imaged with the Typhoon-Trio system and the Image Quant TL software 7.0 (GE Healthcare, Munich, Germany). Sec62, vimentin, and β-actin levels were quantified and normalized to GAPDH.

### Real-time cell proliferation analysis

The xCELLigence SP and DP systems (Roche Diagnostics GmbH, Mannheim, Germany) were used for the real-time analysis of cell proliferation. These systems measure changes of impendance in special plates with micro electrodes covering the well bottoms (E-plates, Roche Diagnostics GmbH, Mannheim, Germany). The relative changes are recorded as Cell Index, a dimensionless parameter. 2.5 × 10^3^ HeLa cells transfected with either siRNA or plasmids were seeded in a 96- or 16-well e-plate (Roche Diagnostics GmbH, Mannheim, Germany) according to the manufacturer’s instructions. Cells transfected with siRNA were seeded 24 h after the second transfection (48 h after the initial siRNA transfection). Cells transfected with plasmids were seeded 24 h after the plasmid transfection. Cell proliferation was monitored for 96 h and the data was evaluated with RTCA 2.0 software (Roche Diagnostics GmbH, Mannheim, Germany). All cell proliferation experiments were repeated fourfold (*n* = 4) and a triplicate of every cell population was analyzed in each experiment.

### Migration potential analysis

Cell migration was analyzed using CIM-devices and the xCELLigence DP system (Roche Diagnostics GmbH, Mannheim, Germany) as a technique of real-time migration monitoring. 2.0 × 10^4^ HeLa cells transfected either with siRNA or plasmids were seeded 24 h after the final transfection in the upper chamber of the CIM-device in culture medium with 5 % FBS. The upper chamber was then placed on the lower part of the CIM-device containing culture medium either supplemented with 10 % FBS as a chemoattractant for cell migration or without FBS (negative control). Cell migration was followed over a time period of 48 h by changes of the impedance signal in the CIM-plate system measured on the backside of the membrane. In parallel, cell proliferation was monitored in a 96-well e-plate (xCELLigence SP system) or in a 16-well e-plate (xCELLigence DP system) as described above.

The BD Falcon FluoroBlok system (BD, Franklin Lakes, NJ, USA) with 8 μm pore inserts for 24-well plates was also used to assess migration. 5 × 10^4^ HeLa cells transfected with either siRNA or plasmids were loaded into the inserts in normal medium containing 5 % FBS. The inserts were then placed in the wells of a 24-well plate in medium with either 10 % FBS as a chemoattractant for migration or without FBS (negative control). After 15 h (39 h after the last transfection), the cells were fixed with methanol, the nuclei counterstained with DAPI and the number of migrated cells was analyzed by a bottom reading fluorescence microscope.

All cell migration experiments were repeated fourfold (*n* = 4) and a triplicate of every cell population was analyzed in each experiment.

### Immunofluorescence of cultured cells

5 × 10^5^ HeLa cells either transfected with *SEC62* siRNA, a *SEC62* plasmid, control siRNA or a control plasmid were seeded onto polylysine coated coverslips. 24 h later, the coverslips were transferred into the wells of a 6-well plate and covered with PBS at 4 °C for 3 min. All following steps were performed in a light protected environment. The cells were fixed in paraformaldehyde for 20 min at 4 °C. The coverslips were then washed four times in PBS (+0.1 M glycine/4 mM MgCl_2_) before incubating with PSS (PBS + 5 % FCS/0.1 % saponine/50 μg/ml RNAse I) for membrane permeabilization and blocking for 1 h. The coverslips were incubated in primary antibody diluted in PSS (1:100 for Sec62-, vimentin- and E-cadherin antibody; 1:250 for Phalloidin-Alexa488 (Life Technologies, Carlsbad, CA USA)) for 1 h, washed twice with PSS, and incubated with secondary antibody diluted 1:1000 in PSS (anti-rabbit Alexa488 and anti-mouse Texas Red; Life Technologies, Carlsbad, CA, USA) before the final three washes in PSS. The coverslips were air-dried and mounted on microscope slides with DAPI-Fluoroshield mounting medium. Imaging was performed as described for IFC.

### Statistical analysis

Statistical analysis of IFC and FISH was performed with a two-sided Mann–Whitney-*U*-test using the Statistical Package for the Social Sciences v. 17.0 (IBM, Chicago, IL, USA) and XLStat Pro (Addinsoft, NY, USA) software. Normality test and statistical analysis of cell proliferation and migration was performed with the D’Agostino & Pearson normality test and a two-sided, paired Student’s *t*-test using GraphPad Prism 6.0 h (GraphPad Software, La Jolla, CA, USA). *P*-values <0.05 were considered statistically significant (α = 0.05). In the figures, statistically significant results are marked by * (*p* ≤ 0.05), ** (*p* ≤ 0.01) or *** (*p* ≤ 0.001). Statistically non-significant results are marked by “n.s.”.

## Results

### The incidence of *SEC62* gains and amplifications rises with the grade of dysplasia

**Fig. 1:**
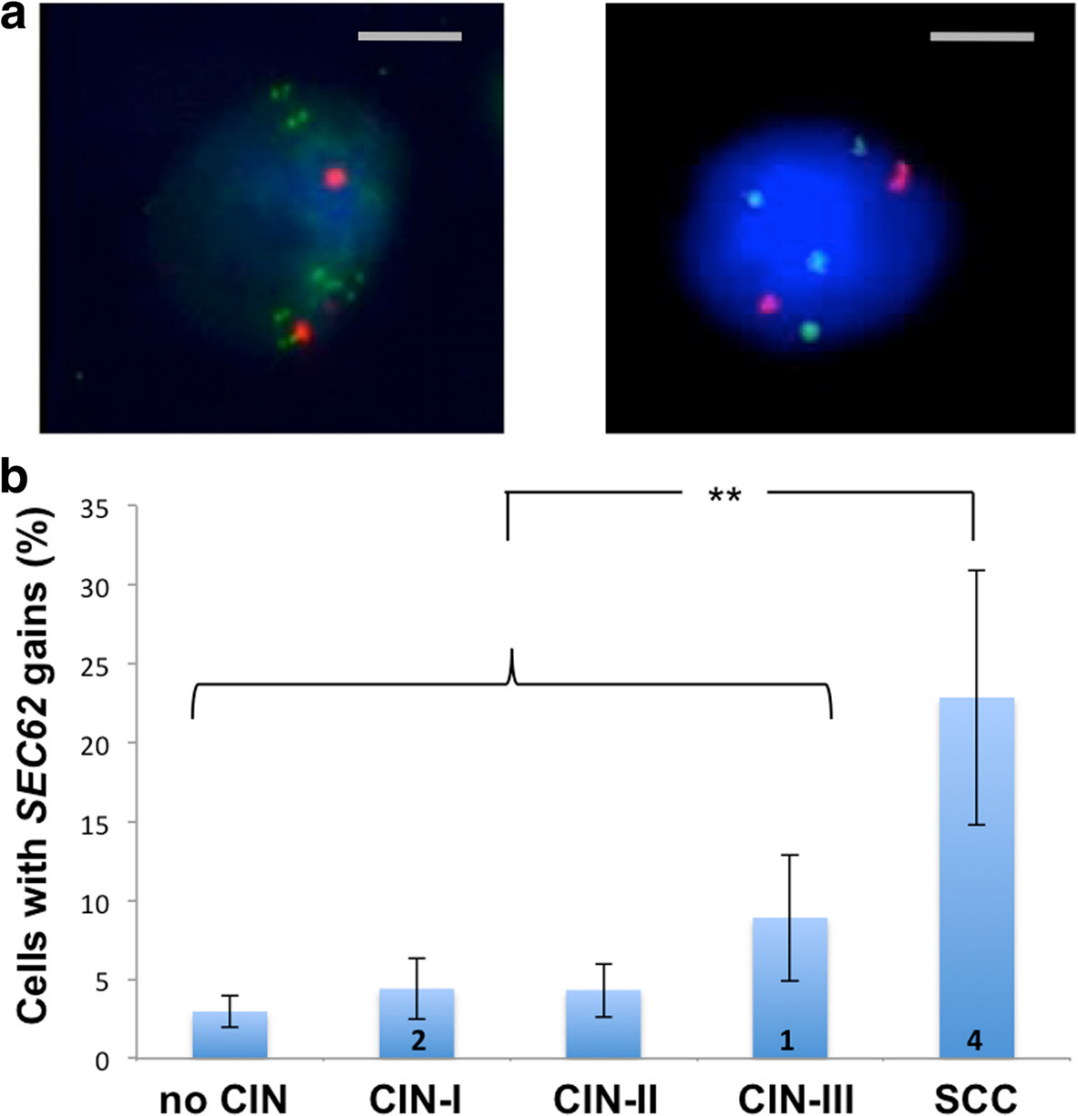
FISH Analysis of dysplastic cervical cells. **a** Fluorescence in situ hybridization (FISH) analysis with a *SEC62*- (*green*) and control chromosome 10 centromere probe (*red*) with representative images of *SEC62* amplifications (*left*) and gains (*right*). **b** The percentage of cells with *SEC62* gains is illustrated by blue bars for the different histomorphological groups (no CIN, CIN-I, CIN-II, CIN-III, SCC). The number of smears showing *SEC62* amplifications is indicated by the number in the respective bar. In total, 5 smears per group were investigated with FISH analysis. The respective standard error is indicated by an error bar. The grey scale bars indicate 10 μm

To determine whether the copy number of the 3q26 encoded *SEC62* gene changes in dysplastic cells of the uterine cervix, we performed FISH analyses of *SEC62* on cytological specimens in a representative subset of 20 patients. Their histological diagnoses were “no CIN” (*n* = 5), CIN-I (*n* = 5), CIN-II (*n* = 5), CIN-III (*n* = 5) and SCC (*n* = 5; for the corresponding cytological diagnoses, see Methods). The centromere region of chromosome 10 served as an internal control. Gains of the *SEC62* gene were found in 3 % of the counted nuclei in normal cases, 4 % of the nuclei in CIN-I cases, 4 % of the nuclei in CIN-II cases, 9 % of nuclei in CIN-III cases and 23 % of nuclei in SCC cases (Fig. 1). Additionally, amplifications of the *SEC62* gene were found in dysplastic nuclei of two CIN-I cases, one CIN-III case and four SCC cases. Overall, we observed a rise of *SEC62* gains and amplifications corresponding to the severity of dysplasia with a significantly higher incidence of *SEC62* gains in SCC cases compared to all other cases (*p* = 0.006).

### Simultaneous overexpression of Sec62 and vimentin designates higher grades of cervical dysplasia

**Fig. 2:**
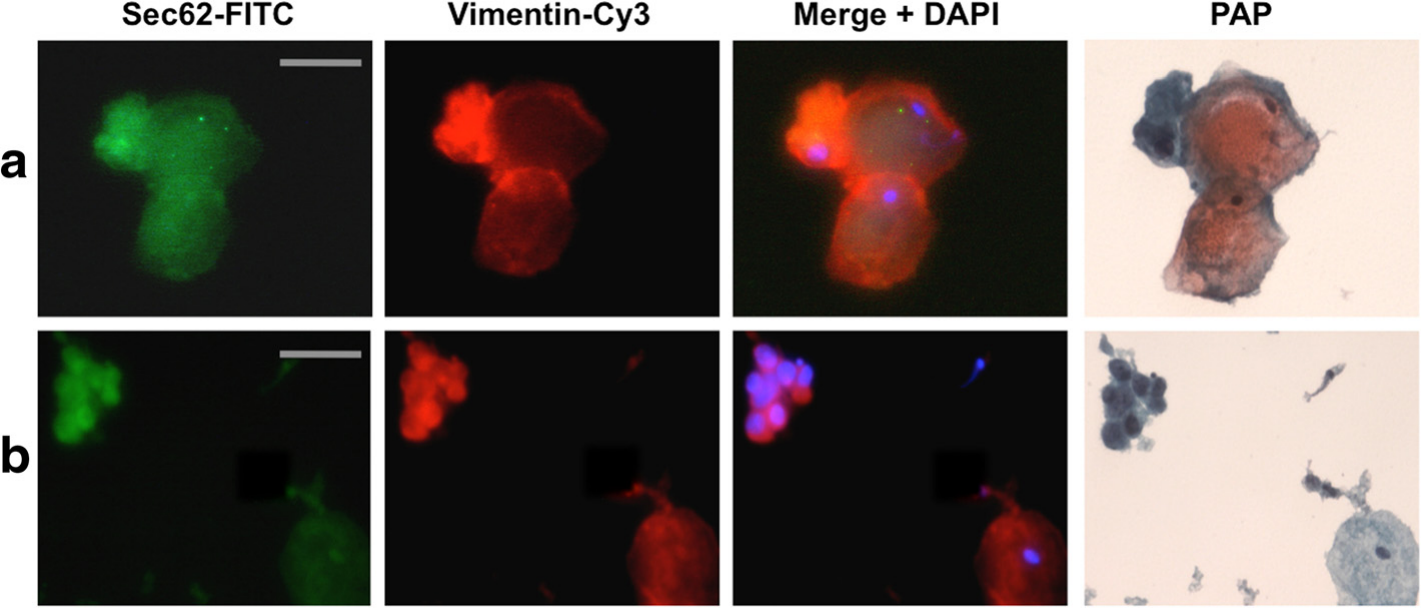
Analysis of *SEC62* and vimentin expression in swabbed cells by immunofluorescence cytology. Sec62 (*left column*, *green*) and vimentin (*middle left column*, *red*) stainings are shown for two representative patients. In the middle right column, both signals are merged and a blue signal indicating the DAPI-stained nuclei is added. Subsequently, the same smears were stained according to Papanicolaou (*right column*) for morphological evaluation of the respective cells and classified according to the Bethesda system as LSIL (**a**) and HSIL (**b**). Cytological images are shown in 100× magnification. The grey scale bars indicate 20 μm

**Fig. 3:**
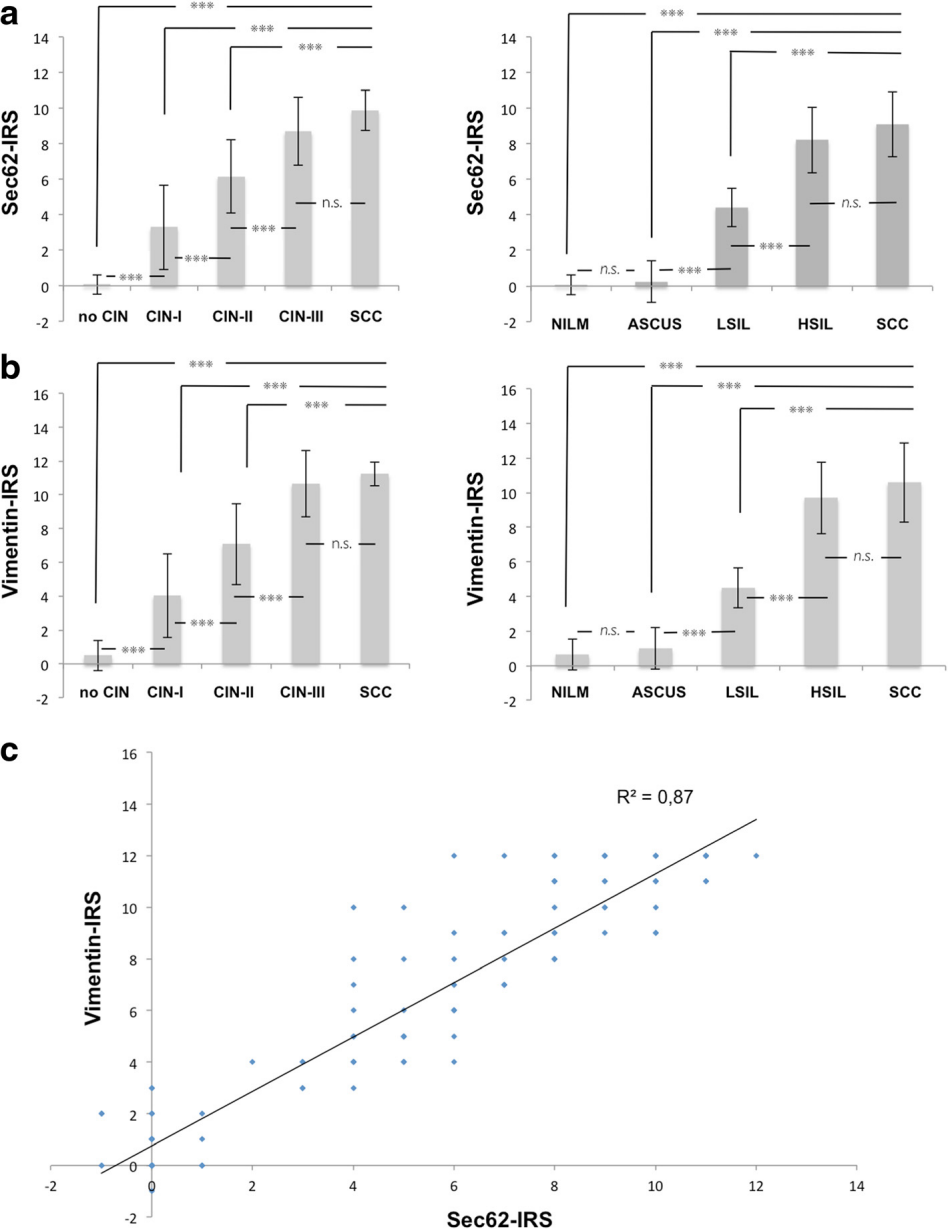
*SEC62* and vimentin expression in dysplastic cervical lesions. IRS for Sec62 (**a**) and vimentin (**b**) immunostaining of uterine cervical smears from 107 women (*n* = 25 + 25 + 25 + 25 + 7 for no CIN, CIN-I, CIN-II, CIN-III, SCC). The cytological immunoreactive score (IRS) values are illustrated for the respective cytomorphological (*right*) and histomorphological diagnoses (*left*). Sec62 and vimentin immunoreactivity of morphologically conspicuous cells was evaluated compared with normal cells of the same smear and valued as weaker (−1), equal (0), slightly more intense (1), moderately more intense (2) or much more intense (3). For each case, the quantitation of 6 independent examiners was toted up to an overall IRS ranging from −6 to 18. In (**c**), the overall IRS for Sec62 was correlated with the overall IRS for vimentin. The strength of squared correlation is indicated by the squared correlation coefficient (R^2^)

To evaluate if the detected *SEC62* gains and amplifications correlate with increased cellular Sec62 protein levels, we quantified the level of Sec62 in the swabbed cells of all 107 female patients. As an overexpression of the *SEC62* gene in HEK293 cells has been reported to induce a rise in vimentin expression, suggesting that *SEC62* mediates EMT induction [25], we analyzed Sec62 and vimentin protein levels simultaneously. Therefore, we developed IFC as a new staining method for liquid-based cytological swabs. After imaging the immunostained cells, the slides were used for Papanicolaou staining to evaluate the morphology of the swabbed cells. Figure 2 shows representative images for two patients, whose cervical swabs were staged LSIL (A) and HSIL (B). Figure 3 summarizes the immunoreactive scores (IRS) for Sec62 and vimentin delineated for the different histological and cytological diagnoses for all included patients.

*SEC62* and vimentin were overexpressed in dysplastic cells compared with normal cells on the same slide with a gradual increase of expression corresponding to the rising severity of the dysplasia. When comparing the expression level of Sec62 and vimentin in the dysplastic cells, we found a distinct correlation between the Sec62- and vimentin-IRS (*r*^2^ = 0.87). To exclude that the dysplastic cells show increased fluorescent signals for all cytoplasmic proteins due to an altered cellular shape instead of a specific overexpression of the respective genes, we performed additional IFC stainings for 10 representative cases targeting Sec62 and β-actin (see Additional file 1). Indeed, there was no relevant change of β-actin expression depending on the severity of dysplasia. Therefore, the rise in Sec62 and vimentin protein levels in the dysplastic cervical cells is likely attributed to a specific overexpression of both genes.

### Altering Sec62 protein levels influences HeLa cell migration

The IFC analyses indicated that *SEC62* overexpression marks the transition from intraepithelial neoplasia to an invasive phenotype. To evaluate whether *SEC62* has potential oncogenic function, we altered Sec62 levels in HeLa cells and evaluated changes in cell migration and proliferation. The experiments were repeated fourfold (*n* = 4) and a triplicate of every cell population was analyzed in each experiment.

**Fig. 4:**
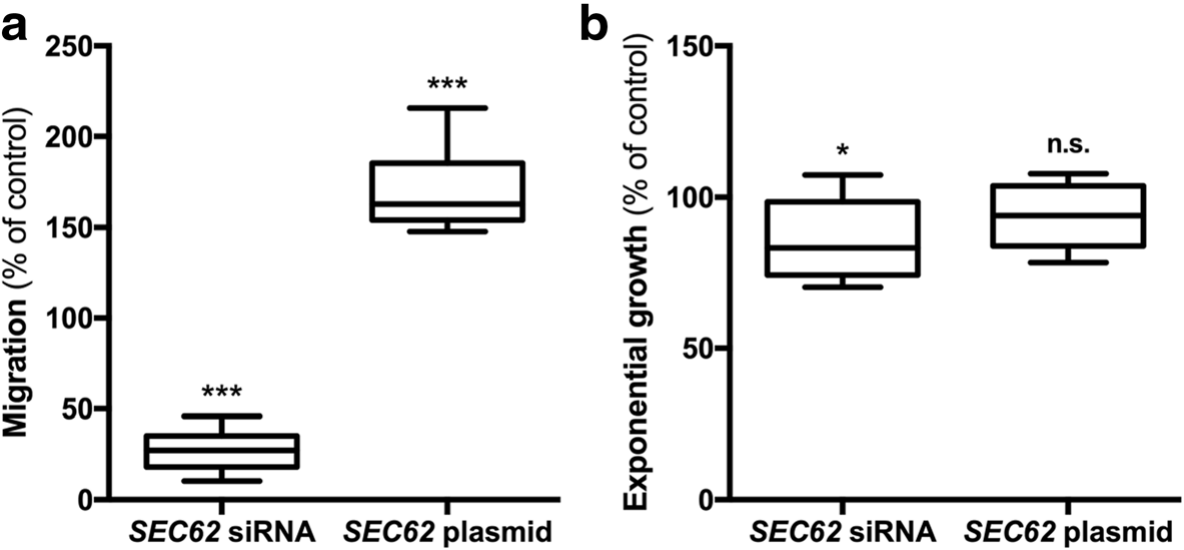
Real-time cell migration (**a**) and proliferation (**b**) analysis of *SEC62*-overexpressing and Sec62-depleted HeLa cells. **a** The cell index was measured as an indicator for migration 15 h after seeding identically pretreated HeLa cells and compared with the respective control cells. **b** The slope of cell proliferation curve was measured during the phase of exponential growth (50–74 h after seeding the cells) for HeLa cells transfected with *SEC62* siRNA or a *SEC62* plasmid and compared with cells transfected with control siRNA or a control plasmid. The experiments were repeated fourfold (*n* = 4) and a triplicate of every cell population was analyzed in each experiment. Cell migration (**a**) and cell proliferation (**b**) are presented as a percentage of the respective controI cells (=100 %) using box and whisker blots. Each box represents the range from the first quartile to the third quartile. The median is indicated by a line. The whiskers outside the boxes represent the ranges from the minimum to the maximum value of each group

**Fig. 5:**
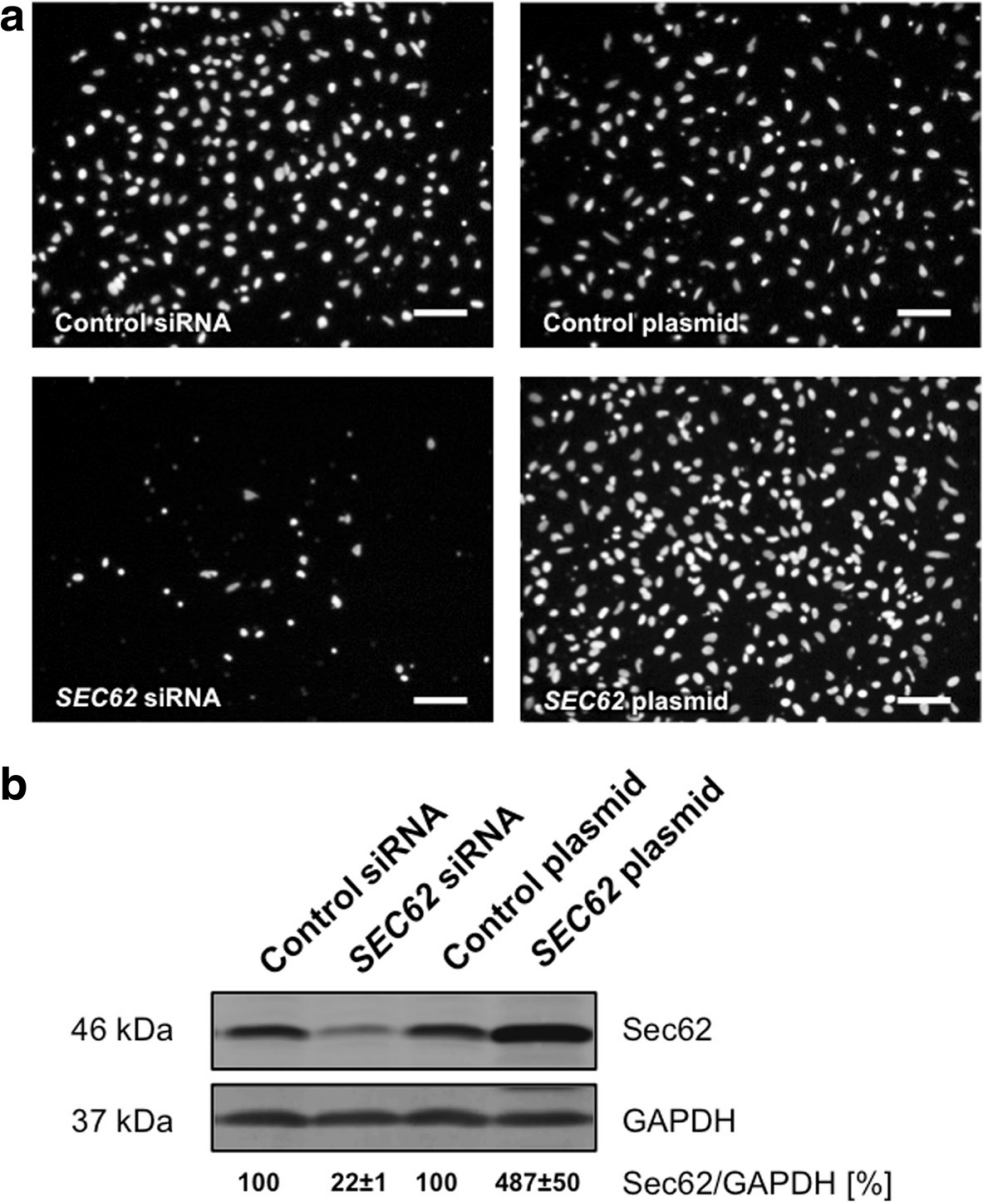
Cell migration analysis of *SEC62*-overexpressing and Sec62-depleted HeLa cells using a trans-well system. The cells that have migrated through the 8 μm sized pores of the insert system were fixed and marked with DAPI (white dots). **a** Representative images are shown for HeLa cells transfected either with control siRNA, *SEC62* siRNA, a control plasmid or a *SEC62* plasmid. **b** Cellular Sec62 protein level of the different cell populations was quantified by western blot and normalized to GAPDH. The relative Sec62 expression is indicated below the respective bands as mean value of 4 identically performed experiments (*n* = 4) with the respective standard error. The white scale bars indicate 100 μm

First, the cells were transfected with *SEC62* siRNA, resulting in decreased Sec62 protein levels to 22 ± 1 % (mean ± standard error of the mean, SEM) compared with control siRNA transfected cells. While marginal effects of *SEC62* silencing on cell proliferation were observed (86 ± 3 %, mean ± SEM), there was a crucial reduction in cell migration (27 ± 4 %, mean ± SEM) compared to control cells using the xCELLigence DP system and the FluoroBlok system for migration monitoring (Figs. 4 and 5).

Next, *SEC62* was overexpressed by transfecting the cells with a *SEC62* plasmid resulting in an increase of Sec62 protein levels to 487 ± 50 % (mean ± SEM) compared with control cells. This overexpression of *SEC62* led to increased cell migration (171 ± 7 %, mean ± SEM) with no influence on cell proliferation (93 ± 3 %, mean ± SEM; Figs. 4 and 5). In all transfection experiments, the transfection procedure itself led to a slightly reduced cell proliferation without however showing relevant differences between the control siRNA and the *SEC62* siRNA transfected cells respectively the control plasmid and the *SEC62* plasmid transfected cells.

### *SEC62* overexpression cannot induce EMT in HeLa cells

**Fig. 6:**
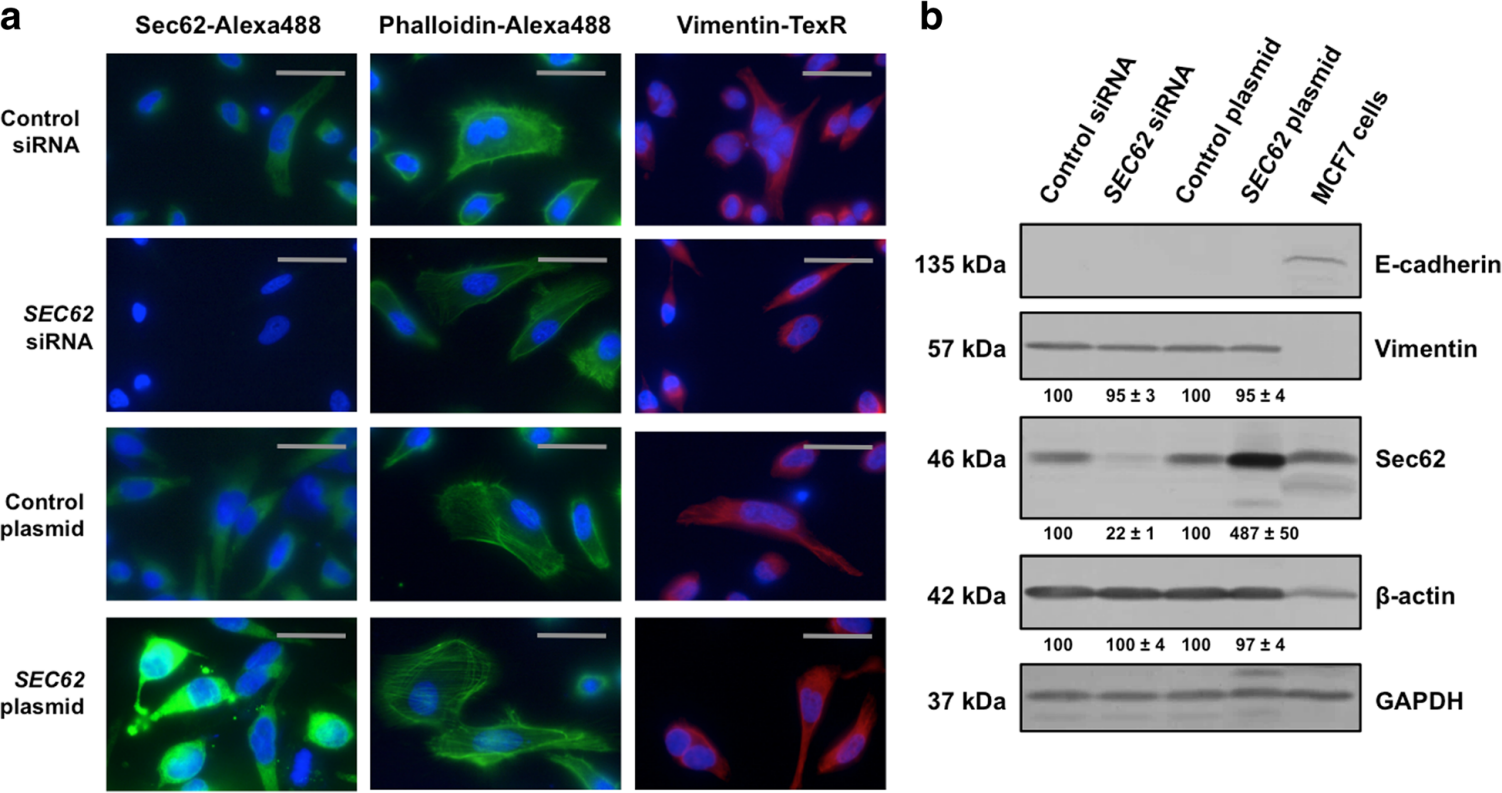
Influence of *SEC62*-overexpression and *SEC62*-silencing in HeLa cells on the expression level of EMT markers. **a** Immunofluorescence targeting Sec62 (left column, green), F-actin (middle left column, green) and vimentin (middle right column, red) in HeLa cells transfected with control siRNA, *SEC62* siRNA, a control plasmid or a *SEC62* plasmid. The nuclei of the cells are marked with DAPI (blue signal). **b** Cellular protein levels of E-cadherin, vimentin, Sec62 and β-actin were quantified in identically pretreated cells and normalized to GAPDH. MCF-7 cells were used as a positive for E-cadherin expression. The relative expression of vimentin, Sec62 and β-actin is indicated below the respective bands as mean value of 4 identically performed experiments (*n* = 4) with the respective standard error. Images in (**a**) are shown in 60× magnification. The grey scale bars indicate 20 μm

As *SEC62* overexpression in HEK293 cells was reported to induce a rise in vimentin expression and a reorganization of the actin cytoskeleton [25], we next investigated if the *SEC62*-driven stimulation of HeLa cell migration can be attributed to an induction of EMT. *SEC62* was either overexpressed by plasmid transfection or downregulated by siRNA transfection and the effects on cellular vimentin and E-cadherin levels were analyzed using western blot and immunofluorescence microscopy. These markers were chosen, because both are known to change their expression level when cancer cells undergo EMT with an upregulation of vimentin and a downregulation of E-cadherin levels [33]. As the subcellular F-actin structure shows structural changes during EMT too [34], we additionally analyzed β-actin as a third EMT marker. There were no changes in the expression level of vimentin, E-cadherin and β-actin and no changes in the subcellular structure of the β-actin cytoskeleton (Fig. 6). All differentially pretreated HeLa cell populations contained a moderate expression of vimentin and β-actin independent of the different treatments and E-cadherin was not detected in HeLa cells agreement with previous reports [35].

## Discussion

Cervical cancer represents the third most common cancer in women worldwide, resulting in approximately 275,000 deaths each year [1]. Despite much effort to develop new diagnostic [36, 37] and therapeutic strategies [38], the 5-year survival rate has remained at about 70 % with no significant changes over the past 30 years [39]. 3q amplification has been identified as a common genomic alteration in cervical cancer [3, 4], marking the transition of intraepithelial neoplasia to invasive cancer [7]. Recently, we observed that *SEC62* encoded at 3q26.2 was frequently amplified and overexpressed in NSCLC tissue specimens [15]. Moreover, a high expression of *SEC62* predicts a poorer clinical outcome for this cancer entity [24] and crucially influences cell migration, calcium homeostasis and ER stress tolerance of various human tumor cells [23, 24, 40].

In this study, we investigated the potential role of *SEC62* in the carcinogenesis of cervical cancer.

We found (i) that *SEC62* is a potential candidate gene of the amplified 3q region in precancerous and early-stage cancerous cervical lesions, (ii) that *SEC62* is overexpressed on the protein level in dysplastic cells of the uterine cervix compared to normal cells and (iii) that the ability of cervical cancer cells’ to migrate depends on their cellular Sec62 protein level.

FISH analyses of representative uterine cervix samples demonstrated a rise in *SEC62* gains and amplifications corresponding to the grade of dysplasia, with the highest incidence in invasive cancer cases. Accordingly, we detected an increase in cellular Sec62 protein level correlating to the severity of dysplasia in IFC analyses. These results agree with previous studies reporting a comparable incidence for the amplification of the entire 3q26 region in precancerous cervical lesions and cervical cancer [41], suggesting that the *SEC62* gene harbors an oncogenic function. However, there are other potential 3q26-encoded oncogenes with a similar pattern of amplification and overexpression in dysplastic cervical lesions including *hTERC*, *LAMP3* and *PIK3CA* [42–45]. Kuglik et al. reported that gains of the *hTERC* gene are specific genomic changes in cytological specimens of the uterine cervix associated with the progression to a malignant phenotype [42]. Furthermore, a meta-analysis of 12 studies evaluating the diagnostic value of *hTERC* in dysplastic cervical lesions found that the detection of *hTERC* amplification is a valuable marker for high-grade cervical lesions and invasive cancer [43]. However, no functional analyses have been performed to confirm the potential oncogenic function of *hTERC* or the molecular mechanism behind its oncogenic activity. It is also probable that multiple genes in the 3q26 region are responsible for the transition of precancerous cervical lesions to invasive cancer and that their interplay bridges the gap from 3q amplification to the molecular cell biology of cervical cancer carcinogenesis.

As in the first part of our study FISH- and IFC-analyses indicated a potential oncogenic function of *SEC62*, we sought to identify a functional correlate in cancer cell biology using HeLa cells an in vitro model. Thereby, *SEC62*-silencing significantly inhibited cell migration while conversely, *SEC62* overexpression stimulated cell migration.

These results confirmed conclusions of previous studies reporting similar effects of *SEC62* gene silencing on lung cancer, prostate cancer, fibrosarcoma, glioblastoma and thyroid cancer cell lines [15, 23], as well as effects of *SEC62* overexpression on human embryonic kidney cells [24]. However, the molecular mechanism of how *SEC62* is able to regulate cell migration remains elusive. *SEC62* encodes for a transmembrane protein of the endoplasmic reticulum (ER) that is thought to be involved in protein transport across the ER membrane, including the translocation of the C-terminus of membrane proteins [20], the membrane insertion and orientation of moderately hydrophobic signal anchor proteins [21] and the secretion of small proteins independent of the signal recognition particle pathway [22]. Hence, we speculate that Sec62 might influence the intracellular transport of proteins that are involved in cell migration.

We previously reported that Sec62 overexpression in HEK293 cells resulted in an increased vimentin expression and observed a structural reorganization of the actin cytoskeleton [25]. As increased vimentin expression is a key marker of EMT [29], *SEC62*-mediated increase of vimentin expression represents an alternative mechanism of how *SEC62* could influence cell migration. In support of this hypothesis, IFC- analyses of cervical brush biopsies demonstrated a distinct correlation between *SEC62* and vimentin expression in our study. However, changes in Sec62 protein levels in HeLa cells did neither result in detectable changes of the expression of EMT markers nor a rearrangement of the actin cytoskeleton structure, contrary to our previous findings in HEK293 cells [25]. A possible explanation for these contradictory results could be that different human cell lines have a varying capability for EMT induction [46] and cytoskeleton remodeling [47]. Alternatively, it is possible that *SEC62* can induce EMT in vivo but requires unknown accessory factors and thus, loses this function in an artificial cell culture model.

Irrespective of the underlying molecular mechanism, the inhibition of cell migration by *SEC62* silencing represents a promising approach for a new targeted therapy, as the molecular effects of *SEC62* silencing on cell migration and ER stress tolerance can be mimicked by trifluoperazine [24], an antipsychotic drug used to treat schizophrenia patients [48]. In addition to this potential role of Sec62 as a therapeutic target, the detection of *SEC62* overexpression by IFC could serve as a potential indicator for 3q26 amplification. As this genomic alteration has a high predictive value for distinguishing CIN-II/III lesions from normal cases [41] and can predict the further development of precancerous cervical lesions [49], Sec62-IFC may provide useful information for the treatment of women with dysplastic cells in their cervical swab.

## Conclusions

Taken together, our study has demonstrated a rising incidence of *SEC62* gains and amplifications in dysplastic cervical lesions as well as an increased cellular Sec62 protein levels corresponding to the severity of dysplasia. In functional analyses, we found that *SEC62* overexpression promoted an invasive phenotype by stimulating the cervical cancer cells’ capability to migrate. Thus, we propose that *SEC62* functions as a migration-stimulating oncogene in the carcinogenesis of cervical cancer and constitutes not only a potential marker for 3q26 amplification but also a potential target for anti-cancer treatment.

## Additional file

Additional file 1:
**Figure S1.** Detection of β-actin (left column) and Sec62 (middle left column) in swabbed cervical cells. In the middle right column, both signals are merged and the right column shows the DAPI-stained nuclei of the cells. The corresponding PAP-stained smears were classified as ASCUS, LSIL and HSIL. Cytological images are shown in 20× magnification. The grey scale bars indicate 50 μm. (TIFF 6165 kb)

## Abbreviations

ASCUS: Atypical squamous cells of undetermined significance
CIN I/II/III: Cervical intraepithelial neoplasia grade I/II/III
EGF: Epithelial growth factor
EMT: Epithelial-mesenchymal transition
ER: Endoplasmic reticulum
FISH: Fluorescence in situ hybridization
HNSCC: Head and neck squamous cell carcinoma
HSIL: High-grade squamous intraepithelial lesion
IFC: Immunofluorescence cytology
IRS: Immunoreactive score
LSIL: Low-grade squamous intraepithelial lesion
NILM: Negative for intraepithelial lesion/malignancy
NSCLC: Non-small cell lung cancer
SCC: Squamous cell carcinoma
