# Supplementary figures and images for: Identification of SEC62 as a potential marker for 3q amplification and cellular migration in dysplastic cervical lesions
Source: BMC Cancer. 2016 Aug 23;16(1):676. doi: 10.1186/s12885-016-2739-6 (PMC4995743; doi:10.1186/s12885-016-2739-6)

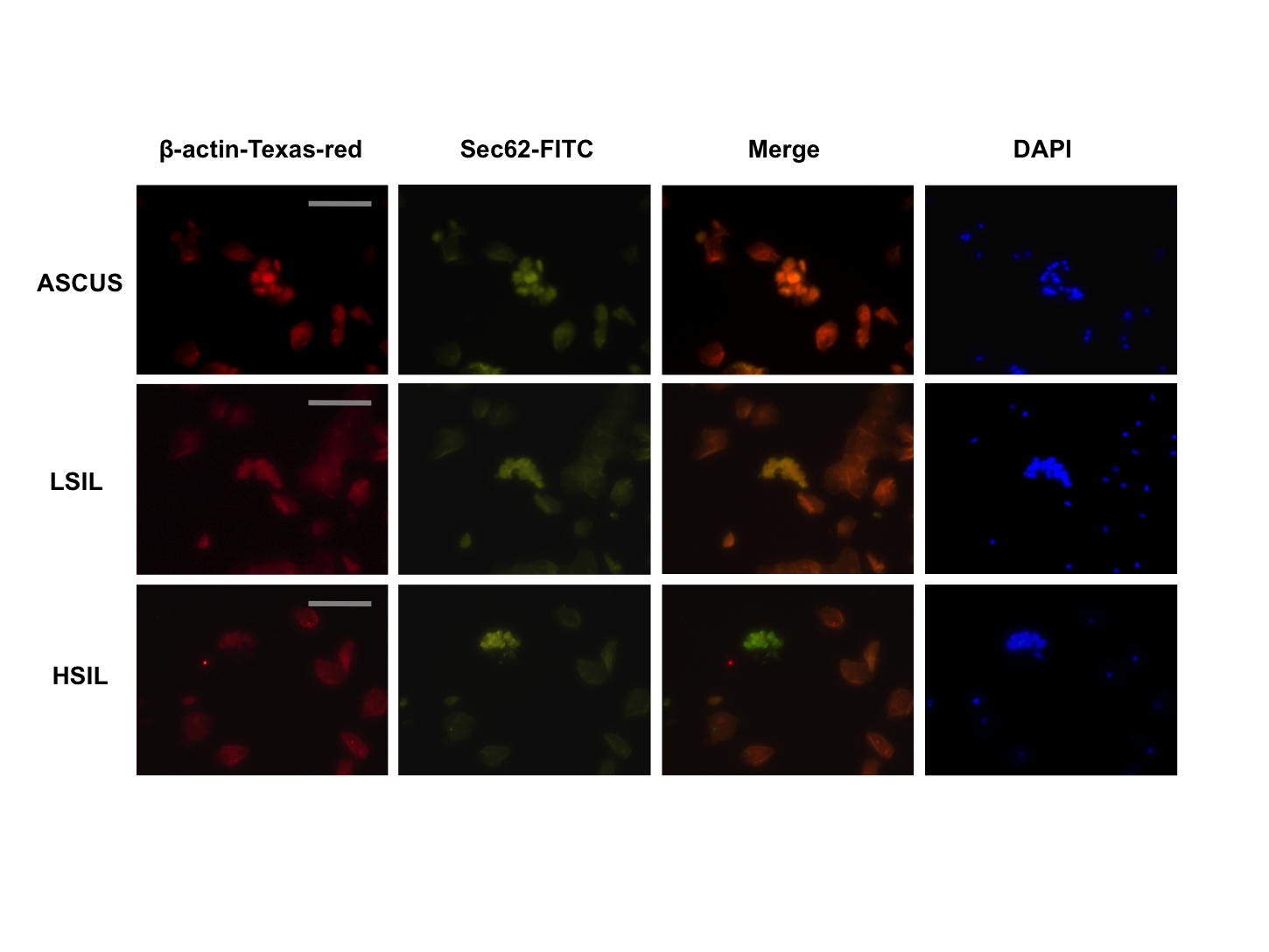

Supplement: Additional file 1: — Figure S1. Detection of β-actin (left column) and Sec62 (middle left column) in swabbed cervical cells. In the middle right column, both signals are merged and the right column shows the DAPI-stained nuclei of the cells. The corresponding PAP-stained smears were classified as ASCUS, LSIL and HSIL. Cytological images are shown in 20× magnification. The grey scale bars indicate 50 μm. (TIFF 6165 kb) [file 12885_2016_2739_MOESM1_ESM.tiff]
